# Insidious Attentional Deficits in Patients With Cerebral Small Vessel Disease Revealed by Attention Network Test

**DOI:** 10.3389/fneur.2022.865307

**Published:** 2022-06-20

**Authors:** Yunliang Guo, Shuo Zhao, Xunyao Hou, Shanjing Nie, Song Xu, Yan Hong, Yali Chen, Shougang Guo, Xueping Liu, Zhangyong Xia

**Affiliations:** ^1^Department of Geriatric Neurology, Shandong Provincial Hospital Affiliated to Shandong First Medical University, Jinan, China; ^2^Department of Neurology, Liaocheng People's Hospital and Liaocheng Clinical School of Shandong First Medical University, Liaocheng, China; ^3^Department of Geriatric Neurology, Shandong Provincial Hospital, Cheeloo College of Medicine, Shandong University, Jinan, China; ^4^Department of Geriatrics, Shandong Provincial Hospital Affiliated to Shandong First Medical University, Jinan, China; ^5^Anti-Aging Monitoring Laboratory, Shandong Provincial Hospital Affiliated to Shandong First Medical University, Jinan, China; ^6^Department of Radiology, Shandong Provincial Hospital Affiliated to Shandong First Medical University, Jinan, China; ^7^Department of Neurology, Shandong Provincial Hospital Affiliated to Shandong First Medical University, Jinan, China; ^8^Department of Neurology, Shandong Provincial Hospital, Cheeloo College of Medicine, Shandong University, Jinan, China; ^9^Department of Neurology, Liaocheng People's Hospital, Cheeloo College of Medicine, Shandong University, Jinan, China

**Keywords:** cerebral small vessel disease, attention, attention network test, alerting, orienting, executive control, total CSVD score

## Abstract

**Background:**

Several reports have indicated potential cognitive decline for cerebral small vessel disease (CSVD), especially in attention domain, whereas the attentional function at network level is still elusive. In this study, we used the attention network test (ANT) paradigm to characterize the efficiency of the alerting, orienting, and executive control networks in patients with CSVD and explore possible correlations between attention network efficiencies and obtained CSVD total score.

**Methods:**

A total of 31 patients with CSVD and 30 healthy controls matched for age, gender, and education level were recruited. After neuropsychological and anxiety/depression/somatization assessments, an original version of ANT containing different cue conditions and target stimuli was used to investigate independent attentional components, and then, behavioral performance (accuracy and reaction time) and network efficacy were recorded and analyzed.

**Results:**

Assessed by traditional neuropsychological scale (MoCA), we did not find difference between groups on general cognition. Nevertheless, the overall reaction time to targets of ANT was markedly prolonged in patients with CSVD, and similar phenomenon was observed for overall accuracy on ANT. Moreover, patients showed significantly lower orienting and executive control network efficiencies compared with controls, while not for alerting network. These impairments were correlated with total CSVD burdens, but not with anxiety, depression, or somatization.

**Conclusions:**

Although general and almost all individual cognitive function evaluated by MoCA seemed to remain intact, the orienting and executive control function was impaired in individuals with CSVD, which was modulated by lesion grades. Our observations implied insidious attentional deficits regarding CSVD. Given this, considering its simplicity and sensitivity, ANT could serve as an attractive tool for early diagnosis of cognitive dysfunction. Further investigations on the availability of ANT detection for CSVD are warranted.

## Introduction

Cerebral small vessel disease (CSVD) denotes a spectrum of clinical, imaging, and pathological syndromes that results from various factors that affect small vessels in the brain, including small arteries, arterioles, capillaries, and venules (1). Accounting for 20–30% of ischemic strokes, its prevalence is about 5% in population aged 50 years, whereas for elderly at 90 years, the incidence rate increases to almost 100% (2). The major manifestations of CSVD visible on magnetic resonance imaging (MRI) comprised lacunes, recent small subcortical infarcts (RSSIs), white matter hyperintensities (WMHs), microbleeds (CMBs), enlarged perivascular space (EPVS), and brain atrophy (3). Most patients are asymptomatic or have mild clinical symptoms, such as lacunar syndrome, urinary incontinence, and gait disorder, whereas subjective cognitive decline is complained by patients with CSVD, which leads to lower quality of life and impaired sociability (4).

There has been evidence indicating diverse degrees of cognitive dysfunction in patients with CSVD (5). Characterized by insidious onset, CSVD is considered as the primary precipitant of vascular cognitive impairment, which could progress into vascular dementia eventually (6). Several traditional neuropsychological assessment tools, such as Mini-Mental State Examination (MMSE) and Montreal Cognitive Assessment (MoCA), were predominantly used in clinical practice (7) and cohort investigation (8). Nevertheless, due to rather limited sensitivity and incapability to detect specific subdomains, these scales might cause possible missed diagnosis, especially for individuals at early stage of mild cognitive impairment (MCI). Up to date, multiple techniques with excellent spatiotemporal resolution and specificity are emerging, such as event-related potential and functional MRI (fMRI), which have been increasingly employed to explore neurocognitive processing in neurological diseases (9, 10). Our recent publication revealed that, although general cognition assessed by MMSE and MoCA was normal, the processing of emotional information detected by event-related potential was impaired in patients with CSVD (11), suggesting that more sensitive neuropsychological assessment methods are necessary in CSVD evaluations.

Attention is a pivotal component of cognition and acts as a fundamental psychological process in enabling further cognitive operations at later processing stages. It allows us to allocate appropriate processing resources to external stimuli, discriminate preferential choices, and optimize performance toward behavioral goals, which is indispensable in daily life (12). As for CSVD, characteristic cognitive decline patterns were observed, with early involvement in attention and executive function domains (7). We also discovered that patients with CSVD had attentional processing deficits regarding visual spatial information detected by event-related potential, without changes in general cognitive function evaluated by MMSE and MoCA, whereas cognitive subdomains were not assessed in the previous study (13). Since attention is regarded as an extremely complex system, it is still in urgent demand to illuminate network-level pathology for CSVD.

Put forward by Posner and Petersen (14), the attention network theory suggested that attention system could be divided into three anatomically and functionally independent components containing alerting, orienting, and executive control, which were regulated with specific cerebral areas and neurotransmitters. The alerting network is responsible for sustaining an internal state when preparing to perceive and respond to stimuli and is mediated by thalamus, prefrontal cortex, and parietal region; the orienting network serves to prioritize sensory inputs by selecting information and shifting attention from one area or object to another, which is mainly modulated with parietal lobe, temporoparietal junction, and frontal eye fields; the executive control network is involved in monitoring and resolving conflicts in situations of error detection and decision-making, and the most associated structure is cingulate cortex and prefrontal cortex (15, 16). Based on the attention taxonomy, Fan and collaborators developed a sensitive evaluation tool, the attention network test (ANT). It is a computerized test that presents a sequence of visual stimuli, which is designed to measure the efficiency of the three attentional networks described above in a single task (17). This reliable tool has been widely applied in sufferers with mild cognitive impairment (e.g., migraine without aura, type 2 diabetes mellitus) (18, 19), whose executive control networks were selectively impaired, and thus, ANT might be used in the detection of insidious attentional deficits in patients with CSVD.

Considering mystery of attention system and simplicity of original ANT paradigm, we aimed to comprehensively investigate the possible effect of CSVD on efficiencies of alerting, orienting, and executive control networks by recording and analyzing behavioral parameters [accuracy and reaction time (RT)]. To better assess insidious attentional disturbances, attention network efficiencies detected in patients with CSVD were compared to those of healthy controls without neurological or chronic disorders, who served as the reference. Since total CSVD score could provide a more complete estimate of CSVD lesions in the brain (20) and modulate cognitive performance (8), we explored potential correlations between attention network efficiencies and total CSVD score to further elucidate this effect. Moreover, individuals with CSVD were inclined to develop anxiety, depression, and somatic symptoms (21, 22), which might have impacts on cognitive function (23, 24), and thus, the associations between cognition and these variables were also evaluated. It was hypothesized that patients diagnosed with CSVD suffered from impairments in attention networks, at least for some components, and these abnormalities might be directly correlated with total CSVD burdens, but not with anxiety, depression, or somatic symptoms.

### Methods

#### Participants and Criteria

From January to October 2021, a total of 38 patients with CSVD (21 men) were enrolled from outpatient and inpatient departments of Shandong Provincial Hospital Affiliated to Shandong First Medical University. We also recruited 35 age-matched healthy controls (18 men) from health management center and local community. They had no history of neurological or systemic diseases (e.g., hypertension, diabetes mellitus).

After necessary neurological examinations by specialized neurologists, all subjects underwent cranial MRI, including T1-weighted imaging, T2-weighted imaging, T2 fluid-attenuated inversion recovery sequence (FLAIR), diffusion-weighted imaging (DWI), and magnetic susceptibility-weighted imaging (SWI), together with magnetic resonance angiography. The inclusion criteria for patients with CSVD were as follows: (1) typical imaging features on MRI, including lacunes, RSSIs, WMHs, CMBs, EPVS, and brain atrophy, which fulfilled diagnostic criteria of CSVD proposed by Wardlaw et al. (3); (2) aged between 45 and 75 years; (3) stable condition and satisfying cooperation throughout the experiment. The exclusion criteria for patients with CSVD were as follows: (1) intracranial or extracranial macrovascular stenosis > 50%; (2) non-acute cortical or subcortical infarct with diameter >15 mm; (3) cardiogenic infarct; (4) non-CSVD-related WMHs, such as multiple sclerosis; (5) intracranial hemorrhage; (6) comorbid with other neurological disorders, such as Parkinson's disease and epilepsy; (7) suffering from systemic disorders, such as tumor and hyperthyroidism; (8) moderate to severe anxiety, depression, or somatic symptoms; (9) drug/alcohol abuse or addiction; (10) poor vision, hearing, or illiterate. As for healthy controls, no abnormal finding was observed on neurological examinations or brain morphology. In addition, all participants were verified to be right-handed without remarkable dysfunction in sensory and motor systems.

Moreover, 7 patients (4 men) were excluded—three for poor cooperation during ANT (overall accuracy <90%), two for incomplete or ambiguous clinical features, and two for moderate anxiety or depression symptomology. Thus, we finally included 31 patients with CSVD (17 men). With regard to controls, 5 subjects (3 men) had to be excluded: four for the lack of neuropsychological or anxiety/depression assessments and the other one for absence of imaging data. So, 30 healthy controls (15 men) were included for further analysis.

The study protocol was approved by the Ethical Committee of Shandong Provincial Hospital Affiliated to Shandong First Medical University (SWYX: No. 2020–232), which was performed according to the principles in the Declaration of Helsinki. All participants signed a written informed consent before commencement of the test.

### General Experimental Procedures

Within the study, standardized interview was performed using a structured questionnaire at initial recruitment, and demographic and clinical characteristics of patients with CSVD were collected, including age, gender, education level, body mass index (BMI), hypertension, diabetes mellitus, hyperlipidemia, and coronary heart diseases. Then, all participants were instructed to undergo neuropsychological (MoCA), anxiety/depression (GAD-7 and PHQ-9), and somatization evaluations (PHQ-15) prior to ANT detection. The cognitive function, anxiety/depression state, and somatic symptomology were obtained and analyzed.

### Imaging Assessments

The MRI scanning was completed at Department of Radiology using a 3.0 T scanner (Siemens Prisma, Germany), and all assessments were conducted by two experienced physicians (SZ and XH) who were familiar with CSVD imaging. They were blinded to cognitive and ANT performance.

#### Individual CSVD Markers

Neuroimaging markers of CSVD were rated according to the Standards for Reporting Vascular Changes on Neuroimaging (STRIVE) consensus criteria (3) and were examined in both hemispheres and totaled across hemispheres.

Based on manual visual rating method, the Fazekas scale was employed to rate severity of WMHs in periventricular and deep regions on T2 FLAIR sequence (25). The periventricular WMHs grades were defined as 0 (absence), 1 (cap), 2 (halo), and 3 (extending into deep white matter); as for foci of deep WMHs, scores were set at 0 (absence), 1 (punctate), 2 (beginning confluence), and 3 (extensive confluence). Lacunes were defined as small and ovoid cavities with diameters of 3–15 mm. They were hypointense on T1 imaging with hyperintense surrounding rims on FLAIR imaging (3). CMBs were defined as punctate foci of hypointensity with diameters <10 mm on SWI and were different from vascular flow voids (26). EPVS was defined as round or linear fluid-filled space with diameter <3 mm on T2-weighted imaging and was rated in basal ganglia and centrum semiovale using a validated four-point scale (0, no EPVS; 1, <10 EPVS; 2, 11–20 EPVS; 3, 21–40 EPVS; 4, >40 EPVS) (27).

#### Total CSVD Score

The total CSVD score is an ordinal scale with the combination of four representative neuroimaging markers and better reflects overall burden of CSVD based on imaging data. This scale ranges from 0 to 4, and 1 point was allocated for presence of the followings: (1) one or more lacunes; (2) periventricular WMHs Fazekas grade 3 or deep WMHs Fazekas grades 2 and 3; (3) one or more CMBs; and (4) moderate to severe EPVS (>10 EPVS) in basal ganglia (20, 28). The interrater reliability testing showed satisfying value (Kappa = 0.91) for CSVD total score, and discrepancies were resolved by consensus.

In this investigation, considering rather low specificity for CSVD imaging and protocols of previous studies (20, 29), brain atrophy was listed as inclusion criteria but was not included in total CSVD score despite being a CSVD marker.

### Neuropsychological Evaluations

For patients with RSSIs, cognitive detections were accomplished after 3 months from onset to minimize the impact of cerebral infarcts.

#### MoCA

Owing to satisfying sensitivity toward suspected MCI, we used MoCA scale to evaluate general and individual cognitive function as described by Huang et al. (30). Multiple domains were covered including visuospatial function, executive function, language, attention, calculation, memory, and orientation. The maximum score is 30 points, with score <26 considered cognitive decline (31).

### Assessments of Anxiety, Depression, and Somatic Symptoms

#### GAD-7 and PHQ-9

The anxiety and depression states were evaluated with The Generalized Anxiety Disorder-7 (GAD-7) and The Patient Health Questionnaire-9 (PHQ-9) scales (32, 33). Briefly, all subjects were invited to respond to a series of questions based on their experience during last 2 weeks, which were rated on a four-point scale, ranging from 0 (“never”) to 3 (“nearly every day”). The cutoff scores of GAD-7 and PHQ-9 were both set at 4 to detect anxiety and depression levels, respectively.

#### PHQ-15

Somatic symptoms were measured by The Patient Health Questionnaire-15 (PHQ-15), which is an efficient self-rating scale comprising 15 items (34). Participants were asked for previous 4 weeks to rate the severity of each symptom as 0 (“not bothered at all”), 1 (“bothered a little”), or 2 (“bothered a lot”), and total scores ≥ 5 represents somatization.

### Stimuli and Procedures of ANT

**Figure 1:**
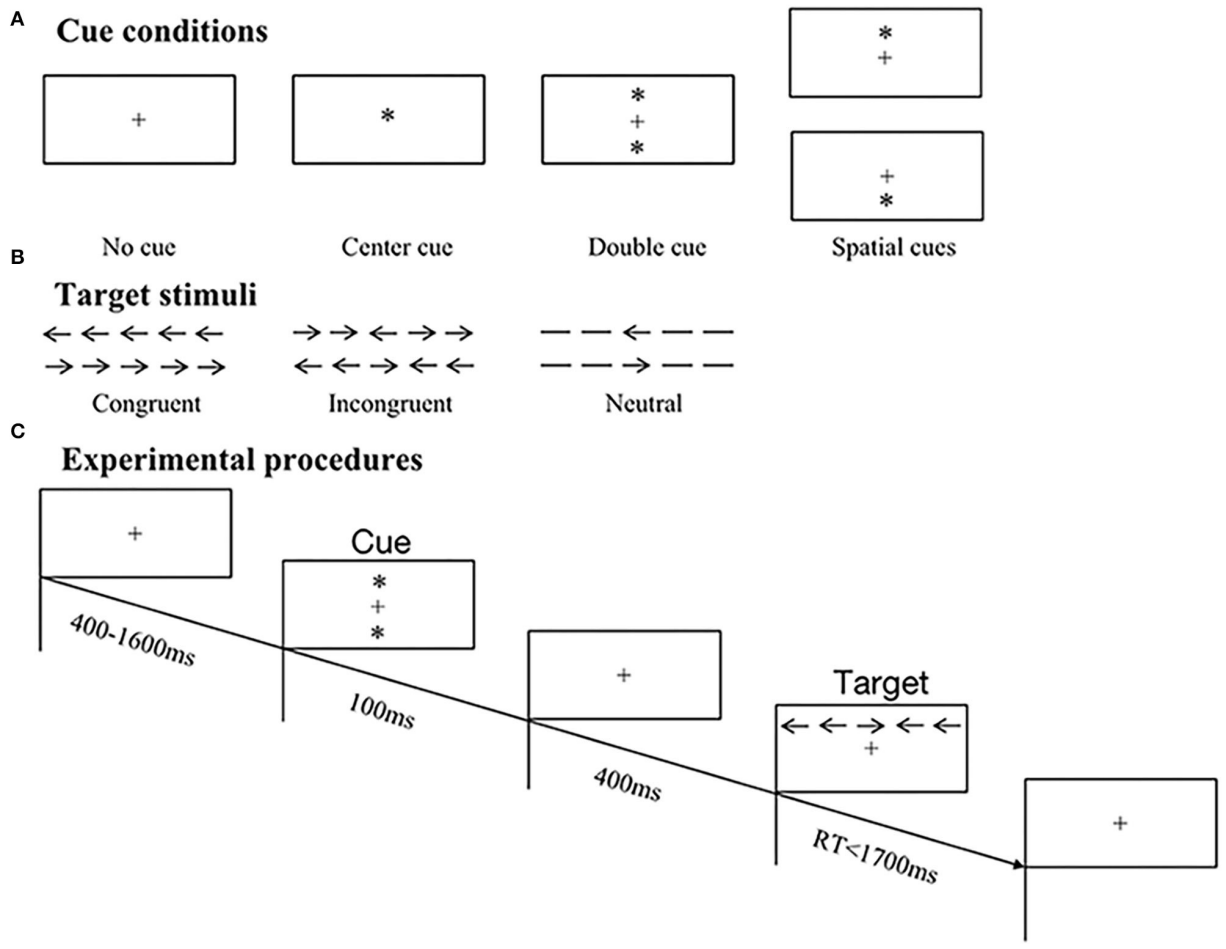
Schematic illustrations of original ANT paradigm. **(A)** Samples of four cue conditions, including no cue, center cue, double cue, and spatial cues. **(B)** Samples of three target types, including congruent, incongruent, and neutral stimuli. **(C)** Experimental procedures of trials in ANT. In this example, a double cue preceded an incongruent target, and the correct response was right button press.

As demonstrated in Figure 1, we used original version of ANT run *via* E-Prime 2.0 software according to the procedures of previous studies (17, 18). The total time taken was ~20 mins.

#### Target Stimuli

The visual target was a leftward or rightward arrow located in the center of five horizontal stimuli and was displayed above or below fixation cross. There existed three types of targets based on the presence and direction of flanking arrows (Figure 1B): (1) congruent–all arrows pointed in the same direction; (2) incongruent–four flanking arrows pointed in opposite direction to the central one; (3) neutral–flankers were replaced by lines without arrowheads (no direction). All subjects were required to discriminate the directions of central arrows and press corresponding left or right mouse button as quickly and accurately as possible.

#### Paradigm and Experimental Procedures

Participants were seated comfortably in an armchair in a sound attenuated chamber and were instructed to fix their eyes at the center of a computer monitor placed in front of them. The original ANT paradigm was employed, which consisted of 12 practice trials providing feedback on whether responses were correct or not. Only if all responses of practice trials were correct could participants move on to experimental tasks. There existed two experimental blocks without feedback, and each block comprised 96 trials displayed in random order, with two repetitions of 48 conditions: 4 cue conditions (no cue/center cue/double cue/spatial cue) × 3 flanker types (congruent/incongruent/neutral) × 2 target directions (left/right) × 2 target locations (above/below). At the beginning of a trial, presentation of fixation cross varied between 400 and 1,600 ms, followed by a series of warning cues lasting for 100 ms and offering invalid or valid information on target positions. Afterward, a fixation cross with duration of 400 ms reemerged prior to the appearance of different target stimuli, and then, targets disappeared to prepare for next trial once response was given within 1,700 ms (refer to Figure 1C).

#### Cue Conditions

Preceding target stimuli, warning cue was represented by an asterisk (“^*^”), which was divided into four conditions based on the presence and position of asterisk (Figure 1A): (1) no cue–a fixation cross (“+”) rather than asterisk was presented; (2) center cue–an asterisk replaced fixation cross, without implying location of upcoming arrays; (3) double cue–two asterisks appeared above and below fixation cross simultaneously, which indicated possible target locations; (4) spatial cue–an asterisk was presented either above or below fixation cross, suggesting the exact position of target stimuli.

#### Preprocessing and Analysis of ANT Data

##### Accuracy

Overall accuracy was calculated as proportion of correct responses within all trials, with value <90% disregarded from analysis.

##### Reaction Time

The RT of correct responses under 12 conditions (4 cue conditions × 3 flanker types) was automatically recorded and further analyzed. To avoid the influence of outliers, an initial data reduction was performed to exclude trials with RT <100 ms or >1,700 ms, and trials with RT exceeding mean ± 2 standard deviations (SD) under each condition (exclusion percentages: patients with CSVD, 6.81 ± 1.09%; healthy controls, 6.61 ± 1.44%; *p* = 0.558 by Student's *t*-test).

##### Attention Network Efficiencies

According to the subtraction method proposed by Fan et al. (17), attention network efficiencies were obtained *via* the following formulae:

(1) Alerting network efficiency = RT_nocue_ – RT_doublecue_

When no cue was presented, participants had maximum temporal uncertainty about occurrence of targets, and thus, it served as control condition of alerting; double cue mostly provided temporal information, elicited alertness to upcoming arrival of target stimuli, and recruited alerting network.

(2) Orienting network efficiency = RT_centercue_ – RT_spatialcue_

The center cue demonstrated no hint about where targets would appear and acted as control condition of orienting; in contrast, spatial cue offered spatial information that allowed subjects to start orienting attention toward target location and activated orienting network.

(3) Executive control network efficiency = RT _incongruent_ – RT_congruent_

Due to conflicting information carried by incongruent targets, more attentional resources in executive control network were allocated under responses to these stimuli when compared with congruent flankers.

Larger values implicated greater efficiencies for alerting and orienting networks; by contrast, for executive control network, its efficiency was lower.

The experimenter was blinded to participants' identity and diagnosis throughout data preprocessing and analysis.

### Statistical Analysis

We used Shapiro–Wilk test to assess the distribution of data. Continuous variables were presented as mean ± SD or median (interquartile range), and categorical variables were expressed as frequency (percentage). Sex ratio between groups was analyzed by χ^2^ test; Student's *t*-test for independent samples or non-parametric Mann–Whitney *U* test was applied in comparisons of other demographic, neuropsychological, and anxiety/depression/somatization characteristics. The mean RT under 12 conditions (4 cue conditions × 3 flanker types) in ANT task was subjected to repeated-measures analysis of variance, with cue (no cue, center cue, double cue, spatial cue) and target (congruent, incongruent, neutral) as within-subject factors, while with group (patients with CSVD vs. healthy controls) as a between-subject factor, and *post-hoc* analysis using Bonferroni correction was conducted if necessary. For attention network efficiencies, Student's *t*-test was employed. Moreover, Spearman's rank correlation coefficients (*r*) were calculated to represent the correlations between attention network efficiencies/MoCA and total CSVD score/anxiety, depression, somatic symptom scores in CSVD group. Statistical calculations were performed with SPSS 23.0 (SPSS Inc., United States). The significance level was set at 0.05 for all analyses, and effect sizes were reported as partial eta squared (η^2^) or Cohen's *d*.

## Results

### Sample Characteristics

Table 1 shows demographic, clinical, and imaging characteristics of subjects. There existed no significant difference between two groups in age, gender, education level, and BMI (all *p* > 0.2). In terms of imaging evaluations, the total CSVD scores were 0 points in 3 (9.68%), 1 point in 12 (38.71%), 2 points in 8 (25.81%), 3 points in 6 (19.35%), and 4 points in 2 (6.45%) patients.

**Table 1:** Demographic, clinical, and imaging characteristics of patients with CSVD and healthy controls.

	**Patients with CSVD**	**Healthy controls**	**Statistical value**	***p-*value**
Number	31	30		
Age, years (range)	60.45 ± 7.47 (46–71)	58.67 ± 7.61 (46–72)	*t*_(59)_ = 0.92	0.359
Gender, male/female	17/14	15/15	*χ^2^* = 0.14	0.705
Education, years	11 (8–12)	12 (9–12)	*Z =* 0.92	0.359
BMI, kg/m^2^ (range)	25.38 ± 2.59 (20.44–31.25)	24.65 ± 2.55 (19.49–30.12)	*t* (59) = 1.10	0.276
Hypertension (percentage)	19 (61.29%)	–		
Diabetes mellitus (percentage)	10 (32.26%)	–		
Hyperlipidemia (percentage)	17 (54.84%)	–		
Coronary heart disease (percentage)	7 (22.58%)	–		
Total CSVD score	2 (1–2.5)	–		

*Continuous variables were expressed as mean ± SD or median (interquartile range)*.

*Categorical variables were expressed as n (%)*.

*Student's t-test was used in analyses of age and BMI*.

*Mann–Whitney U test was used in analysis of education*.

*χ^2^ test was used in analysis of gender*.

*BMI, body mass index*.

As demonstrated in Table 2, although scores of executive function seemed to be lower in patients with CSVD (*Z* = 2.09, *p* = 0.036), the analyses considering global cognitive function assessed by MoCA, as well as other subdomains did not reach significant levels (*p*s > 0.05). No notable distinction was found for GAD-7, PHQ-9 and PHQ-15 scales either.

**Table 2:** Neuropsychological and anxiety/depression/somatic symptom assessments of patients with CSVD and healthy controls.

	**Patients with CSVD**	**Healthy controls**	***Z* value**	***p-*value**
MoCA	28 (25–29)	28 (27–29)	1.70	0.090
Visuospatial function	4 (3–4)	4 (3–4)	0.83	0.405
Executive function	7 (7–8)	8 (7–8)	2.09	0.036^*^
Language	5 (5–6)	5 (5–6)	0.00	1.000
Attention and concentration	3 (2–3)	3 (3–3)	1.70	0.089
Calculation	3 (3–3)	3 (3–3)	1.15	0.250
Memory	4 (3–5)	5 (4–5)	1.05	0.292
Orientation	6 (6–6)	6 (6–6)	0.43	0.671
GAD-7	2 (1–5)	2 (2–3)	−0.57	0.570
PHQ-9	3 (1–4)	2.5 (2–3)	−0.80	0.422
PHQ-15	3 (1.5–4.5)	2 (2–3)	−1.25	0.213

*Data were expressed as median (interquartile range)*.

*Visuospatial function, executive function, language, attention and concentration, calculation, memory as well as orientation are subdomains of MoCA*.

*Visuospatial function refers to copy cube and draw clock tests*.

*Executive function refers to alternating trail making, abstraction, verbal fluency, copy cube and draw clock tests*.

*Language refers to naming, sentence repetition and verbal fluency tests*.

*Attention and concentration refer to vigilance and digit span tests*.

*Calculation refers to serial 7s test*.

*Memory refers to delayed recall and verbal fluency tests*.

*Orientation refers to temporal and spatial orientation tests*.

* *P < 0.05 by Mann-Whitney U test (two-tailed) (indicated as bold)*.

*MoCA: Montreal Cognitive Assessment; GAD-7. The Generalized Anxiety Disorder-7; PHQ-9, The Patient Health Questionnaire-9; PHQ-15, The Patient Health Questionnaire-15*.

### Characterization of ANT Data

#### Accuracy

The overall accuracy was 97.92% (95.31–98.96%) and 98.44% (98.44–98.96%) for patients with CSVD and healthy controls, respectively, and analysis reached significant level (*Z* = 2.27, *p* = 0.023 by Mann–Whitney *U* test; **Table 4**).

#### RT

The overall RT was significantly prolonged for patients with CSVD (778.47 ± 115.17 ms) than that for control counterparts [620.29 ± 69.44 ms; *t*_(49.54)_ = 6.52, *p* < 0.001 by Student's *t*-test; **Table 4**].

The mean RT under each condition in two groups is shown in Table 3. The main effect of group was significant [*F*_(1, 59)_ = 41.97, *p* < 0.001, partial η^2^ = 0.42], indicating that mean RT was markedly delayed in patients with CSVD compared with healthy controls. Across groups, target stimuli were verified to modulate RT [*F*_(2, 118)_ = 457.70, *p* < 0.001, partial η^2^ = 0.89]. Further *post-hoc* analyses revealed that incongruent flankers (767.86 ± 135.30 ms) elicited longer RT than congruent (675.91 ± 125.47 ms) and neutral types (662.16 ± 123.24 ms; both *p* < 0.001), and so did congruent flankers compared with neutral ones (*p* = 0.005). Additionally, there existed remarkable group × target interaction [*F* (2,118) = 14.65, *p* < 0.001, partial η^2^ = 0.20], and subsequent comparisons displayed that patients exhibited prolonged RT toward three flankers [*F*_(1, 59)_ = 33.74, *p* < 0.001, partial η^2^ = 0.36 for congruent targets; *F*_(1, 59)_ = 54.19, *p* < 0.001, partial η^2^ = 0.48 for incongruent targets; *F*_(1, 59)_ = 35.67, *p* < 0.001, partial η^2^ = 0.38 for neutral targets]. We obtained noticeable cue effect [*F*_(3, 177)_ = 91.57, *p* < 0.001, partial η^2^ = 0.61], among which the no cue condition demonstrated longest RT (725.30 ± 131.06 ms), followed by center (712.38 ± 136.96 ms) and double cues (690.94 ± 134.73 ms), whereas RT elicited by spatial cues was fastest (679.28 ± 138.60 ms). It interacted with group [*F*_(3, 177)_ = 3.26, *p* = 0.023] as well, suggesting that patients produced delayed responses compared with controls under each warning cue (all *p* < 0.001). Other analyses did not reach significant levels (*p*s > 0.1).

**Table 3:** Mean reaction time (ms) under each condition for patients with CSVD and healthy controls.

**Groups**	**Target stimuli**	**Cue conditions**			
		**No cue**	**Center cue**	**Double cue**	**Spatial cue**
Patients with CSVD	Congruent	772.47 ± 113.02	753.71 ± 134.38	739.39 ± 116.35	726.91 ± 125.14
	Incongruent	876.56 ± 112.13	860.60 ± 120.55	855.05 ± 118.31	837.49 ± 123.20
	Neutral	758.99 ± 115.49	742.64 ± 128.62	713.46 ± 111.22	721.45 ± 121.15
Healthy controls	Congruent	621.90 ± 73.28	614.24 ± 77.22	589.50 ± 74.06	579.50 ± 71.97
	Incongruent	695.60 ± 74.55	697.68 ± 76.45	664.91 ± 74.48	643.09 ± 74.28
	Neutral	618.55 ± 70.96	598.09 ± 71.46	575.49 ± 68.08	559.00 ± 71.30

*Data were expressed as mean ± SD*.

#### Attention Network Efficiencies

As depicted in Table 4, patients with CSVD displayed remarkably lower efficiencies of orienting [patients, 23.70 ± 10.58 ms; controls, 42.81 ± 12.92 ms; *t*_(59)_ = −6.33, *p* < 0.001, Cohen's *d* = −1.62] and executive control networks [patients, 109.31 ± 26.01 ms; controls, 74.03 ± 14.27 ms; *t*_(46.88)_ = 6.59, *p* < 0.001, Cohen's *d* = 1.68]. No difference was observed for alerting network [patients, 33.38 ± 12.40 ms; controls, 35.38 ± 13.06 ms; *t*_(59)_ = −0.62, *p* = 0.540, Cohen's *d* = −0.16].

**Table 4:** Attention network efficiencies of patients with CSVD and healthy controls.

**ANT parameters**	**Patients with CSVD**	**Healthy controls**	**Statistical value**	***p-*value**
Alerting (ms) (range)	33.38 ± 12.40 (7.51–61.86)	35.38 ± 13.06 (11.81–65.29)	*t*_(59)_ = −0.62	0.540
Orienting (ms) (range)	23.70 ± 10.58 (2.42–43.59)	42.81 ± 12.92 (20.08–75.69)	*t*_(59)_ = −6.33	<0.001^***^
Executive control (ms) (range)	109.31 ± 26.01 (70.37–169.19)	74.03 ± 14.27 (44.88–115.88)	*t*_(46.88)_ = 6.59	<0.001^***^
Overall RT (ms) (range)	778.47 ± 115.17 (570.45–983.26)	620.29 ± 69.44 (467.27–754.96)	*t*_(49.54)_ = 6.52	<0.001^***^
Overall accuracy (%)	97.92 (95.31–98.96)	98.44 (98.44–98.96)	*Z* = 2.27	0.023^*^

*Data were expressed as mean ± SD or median (interquartile range)*.

* *P < 0.05 by Mann–Whitney U test*;

*** *P < 0.001 by Student's t-test (all indicated as bold)*.

*ANT, attention network test; RT, reaction time*.

### Correlations Between Attention Network Efficiencies/MoCA and Total CSVD Score/Anxiety, Depression, Somatic Symptom Scores

We observed significant and moderate relationships between efficiencies of three networks and total CSVD score (*r* = −0.40, *p* = 0.025 for alerting; *r* = −0.40, *p* = 0.026 for orienting and *r* = 0.53, *p* = 0.002 for executive control, respectively), whereas overall RT, overall accuracy, and scores of MoCA/MoCA subdomain executive function did not correlate with CSVD total burdens or anxiety/depression/somatization characteristics (*p*s > 0.05, Table 5).

**Table 5:** Correlations between attention network efficiencies/MoCA and total CSVD score/anxiety, depression, somatic symptom scores in patients with CSVD.

	**Total CSVD score**	**GAD-7**	**PHQ-9**	**PHQ-15**
**Neurocognitive data**	***r* (*p* value)**	***r* (*p* value)**	***r* (*p* value)**	***r* (*p* value)**
Alerting network efficiency	−0.40 (0.025)^*^	−0.07 (0.725)	−0.09 (0.635)	−0.22 (0.231)
Orienting network efficiency	−0.40 (0.026)^*^	−0.03 (0.874)	0.08 (0.688)	−0.32 (0.082)
Executive control network efficiency	0.53 (0.002)^**^	0.31 (0.089)	0.03 (0.859)	0.17 (0.353)
Overall RT	0.18 (0.340)	−0.08 (0.666)	0.07 (0.718)	0.08 (0.675)
Overall accuracy	0.01 (0.949)	0.04 (0.829)	−0.25 (0.167)	−0.12 (0.521)
MoCA	−0.24 (0.186)	−0.19 (0.296)	−0.30 (0.101)	−0.35 (0.053)
Executive function	−0.09 (0.617)	−0.31 (0.092)	−0.19 (0.309)	−0.26 (0.166)

* *P < 0.05*,

** *P < 0.01 by Spearman's correlations (two-tailed) indicated as bold*.

*GAD-7, The Generalized Anxiety Disorder-7; PHQ-9, The Patient Health Questionnaire-9; PHQ-15, The Patient Health Questionnaire-15; MoCA, Montreal Cognitive Assessment*.

## Discussion

In this study, cognitive function in patients with CSVD was evaluated using the MoCA and attentional networks were assessed using the ANT paradigm. Although general cognition and anxiety/depression scores were comparable to healthy controls, behavioral performance (overall RT and accuracy) was impaired on ANT task, indicating abnormalities in attentional networks for individuals with CSVD. In addition, compared with controls, efficiencies of orienting and executive control networks were significantly lower in patients, while not for alerting network, and these markers were correlated with total CSVD score. The aforementioned findings implicated that patients with CSVD suffered from attention network dysfunction, which could be potentially modulated by CSVD imaging markers.

Involved in input selections and attentional shifts, covert orienting is mainly mediated by parietal lobe, temporoparietal junction, frontal eye fields, and pulvinar (35). The cholinergic neurotransmission plays a crucial role in regulation of this activity (36). Although no difference was obtained in attention subdomain score of MoCA (Table 2) relative to healthy controls, the orienting efficiency was markedly attenuated in patients with CSVD (Table 4). There exists several indirect evidence supporting our observation. It was demonstrated that functional connectivity of parietal subregions with cortex regions was significantly different between patients with WMHs and healthy controls, suggesting the disturbances of brain areas related to orienting and possible cognitive decline (37). Additionally, Pedro et al. discovered that patients with MCI with subcortical vascular damage exhibited smaller orienting effect compared with patients free from subcortical vascular damage and controls (38), and subcortical lesions caused by RSSIs and deep WMHs are prevalent for CSVD. Nevertheless, *via* a lateralized ANT-revised paradigm, Cao et al. reported that orienting efficiency of right hemisphere was higher in CSVD group, which could be explained by adaptive compensation hypothesis (39). The divergencies might arise from distinct ANT stimuli and individual criteria for participant enrolment. So, the same ANT version, more homogeneous inclusion criteria, and standardized protocols on measurements of CSVD should be emphasized in the future researches. More importantly, since cholinergic deficit has been verified in postmortem CSVD brains (40), the less efficient orienting might be due to dysfunction of acetylcholine system.

Attentional processes are linked to executive functioning. As the third component of attention network, executive control overcomes task conflicts among competing thoughts and is within broad executive function construct. This network is closely associated with frontal regions, including prefrontal lobe, anterior cingulate cortex, and basal ganglia (41), which are the target areas of dopaminergic circuits (16). There existed evidence that executive function was generally affected in CSVD and vascular cognitive impairment (39, 42). In this investigation, lower executive control efficacy was observed for patients (Table 4). Notably, Lu et al. (43) found that Chinese elderly with cerebrovascular disease had poorer executive attention effects than controls after correcting for general slowing. Similar phenomenon was replicated in subcortical stroke using modified ANT, and pathways connecting prefrontal areas to other regions were disrupted and displayed correlations with prolonged RT of executive control (44). These available publications provide valuable hints for result interpretations regarding CSVD. Corroborating our finding, a recent neuroimaging study revealed that participants with higher burden of WMHs were inclined to develop mild Parkinsonian signs, followed by reduced functional connectivity of cortico-striatal executive networks, which were dopamine-dependent (45). Considering heterogeneity of CSVD imaging, the mechanisms contributing to dysregulation of executive control function deserve full elucidation.

In terms of alerting network, it is activated when warning information precedes target signals, and the resulting phasic alteration facilitates responses to incoming stimuli. Under modulation of noradrenaline system, the most relevant structures for alertness are thalamus, prefrontal cortex, and parietal region, particularly in right hemisphere (16, 46). A comprehensive review has demonstrated that benefits of temporal alerting cues diminish in normal aging rather than pathological cognitive decline, which is owing to lack or insufficiency of compensatory process (47). In line with this attentional pattern, we did not obtain the difference in alerting component between two groups of age-matched subjects (Table 4). Interestingly, alerting-specific areas (e.g., thalamus) are vulnerable to ischemia (48), which might be prevented against damage in CSVD population. Thus, preservations of alerting and responsible regions for patients with CSVD need to be further validated, perhaps in a larger cohort.

Furthermore, a number of Spearman's correlations were calculated for the purpose of exploration and hypothesis generation. Since it has been demonstrated that even subclinical levels of anxiety, depression, and somatization may affect cognitive processing (23, 24, 49), whether cognitive alterations in patients with CSVD were anxiety/depression/somatization-driven needed to be illuminated. No correlation was found between attention network efficiencies and anxiety/depression/somatization characteristics, and the influence of anxiety, depression, and somatic symptoms on these parameters was ruled out. Meanwhile, we discovered that total CSVD score was significantly correlated with attention network efficiencies as measured by the ANT (Table 5), but not with global cognition as measured by the MoCA scores or with executive function as measured by scores on this MoCA subdomain, indicating that higher total CSVD loads would lead to more severe impairments in orienting and executive control, together with providing indirect evidence that the sensitivity of ANT was promising. Likewise, it was verified in our investigation that WMHs grade could affect magnitude and speed of attentional processing (13), and negative relationship between orienting efficiency of right hemisphere and WMHs lesions was observed for patients with CSVD in another study (39). Since total CSVD score was related to cognitive decline and incident dementia (8, 29), the correlations reported in this study highlighted possible damage of brain regions responsible for attention networks in patients with CSVD and modulation of total lesion burdens; hence, early diagnosis and treatment for CSVD might mitigate brain pathology associated with cognitive deficits.

To our knowledge, this was the first study to investigate attention network efficiencies in patients with CSVD by employing original ANT paradigm, and we validated corresponding dysfunction and proposed possible explanations. The profound subject selection criteria and collection of detailed information were included in the strengths of current research. Moreover, ANT-related data were rather homogenate, resulting in substantially greater statistical power. The potential correlations between attention network efficiencies and total CSVD burdens were also systemically discussed and clarified. Nevertheless, there were several limitations constraining interpretation of observations. First, ANT recording was not blinded to examiners, which might cause potential bias of results. In this experiment, an examiner (YG) engaged in data preprocessing and analysis was blinded regarding subjects' identity and diagnosis, which could diminish bias to minimum extent and make conclusions more convincing. Second, the small sample size confined the investigations of age and gender effects, and distinctions among imaging subtypes were not explored either. Moreover, since general and individual cognition was screened by MoCA alone, neuropsychological assessments in this study were limited, and we aimed to reevaluate cognitive function of patients with CSVD more thoroughly by other comprehensive tests (e.g., Stroop Color and Word Test, verbal fluency test) in future investigation to evaluate different aspects of cognition with more extensive and specific assessments, thus, effects of CSVD on cognitive function could be better assessed. Finally, confined by manual visual rating, regional variability of CSVD markers was not evaluated in patients, which would be further measured by more sensitive method (e.g., automatic segmentation rating). In addition, examination of possible associations between location and ANT performance, together with event-related fMRI localization, should be conducted in the near future to uncover exact mechanisms underlying network-level alterations in individuals with CSVD.

Notwithstanding existed limitations, we elucidated that patients with CSVD suffered from impairments in attentional networks subserving orienting and executive control function but were not impaired on the specific cognitive domains assessed by the MoCA screener. These abnormalities were directly correlated with total CSVD burdens, which implied modulatory effect of lesion severity. Our findings shed light on the better understanding of insidious attentional disturbances in CSVD and superiority of ANT detection. To facilitate outcomes for individuals with CSVD, it is important to take cognitive decline into account, particularly in early or subclinical phase, and ANT could be a sensitive and promising tool for neurocognitive assessments.

## Data Availability Statement

The raw data supporting the conclusions of this article will be made available by the authors, without undue reservation.

## Ethics Statement

The studies involving human participants were reviewed and approved by Ethical Committee of Shandong Provincial Hospital Affiliated to Shandong First Medical University (SWYX: No. 2020-232). The patients/participants provided their written informed consent to participate in this study.

## Author Contributions

YG, SZ, XH, SN, SX, YH, and YC performed this study. YG participated in experimental design, data preprocessing, statistical analysis, and manuscript drafting. SZ and XH analyzed imaging data, prepared figures, and aided in revising manuscript for intellectual content. SN and SX helped to enroll subjects, collected needed information, conducted neuropsychological evaluations and carried out ANT detections, with assistances of YH and YC. XL, ZX, and SG cooperated in study design, participant recruitment, discussion of results and manuscript revision. All authors contributed to the article and approved the submitted version.

## Funding

This work was supported by grants from Key Research and Development Project in Shandong Province [No. 2019GSF108101 (XL)], Foundation of Youth Talent of Shandong Provincial Hospital (YG), Incubation Foundation of Shandong Provincial Hospital [No. 2020FY028 (YG)], and the Natural Science Foundation of Shandong Province [No. ZR2020MH139 (ZX)]. All funders provided necessary experimental materials and publication fees.

## Conflict of Interest

The authors declare that the research was conducted in the absence of any commercial or financial relationships that could be construed as a potential conflict of interest.

## Publisher's Note

All claims expressed in this article are solely those of the authors and do not necessarily represent those of their affiliated organizations, or those of the publisher, the editors and the reviewers. Any product that may be evaluated in this article, or claim that may be made by its manufacturer, is not guaranteed or endorsed by the publisher.
